# Unraveling Anisotropy in Crystalline Orientation under Shock-Induced Dynamic Responses in High-Entropy Alloy Co_25_Ni_25_Fe_25_Al_7.5_Cu_17.5_

**DOI:** 10.3390/nano13172446

**Published:** 2023-08-29

**Authors:** Yongchao Wu, Jianli Shao

**Affiliations:** 1 State Key Laboratory of Explosion Science and Technology, Beijing Institute of Technology, Beijing 100081, China; yongchao_wu@bit.edu.cn; 2 Explosion Protection and Emergency Disposal Technology Engineering Research Center of the Ministry of Education, Beijing 100039, China

**Keywords:** shock response, high-entropy alloy, phase transformation, spall damage, molecular dynamics

## Abstract

Shock-induced plastic deformation and spall damage in the single-crystalline FCC Co_25_Ni_25_Fe_25_Al_7.5_Cu_17.5_ high-entropy alloy (HEA) under varying shock intensities were systematically investigated using large-scale molecular dynamics simulations. The study reveals the significant influence of crystalline orientation on the deformation mechanism and spall damage. Specifically, the shock wave velocities in the [110] and [111] directions are significantly higher than that in the [001] direction, resulting in a two-zone elastic-plastic shock wave structure observed in the [110] and [111] samples, while only a single-wave structure is found in the [001] sample. The plastic deformation is dominated by the FCC to BCC transformation following the Bain path and a small amount of stacking faults during the compression stage in the [001] sample, whereas it depends on the stacking faults induced by Shockley dislocation motion in the [110] and [111] samples. The stacking faults and phase transformation in the [001] sample exhibit high reversibility under release effects, while extensive dislocations are present in the [110] and [111] samples after release. Interestingly, tension-strain-induced FCC to BCC phase transformation is observed in the [001] sample during the release stage, resulting in increased spall strength compared to the [110] and [111] samples. The spall strength estimated from both bulk and free surface velocity history shows reasonable consistency. Additionally, the spall strength remains stable with increasing shock intensities. The study discusses in detail the shock wave propagation, microstructure change, and spall damage evolution. Overall, our comprehensive studies provide deep insights into the deformation and fracture mechanisms of Co_25_Ni_25_Fe_25_Al_7.5_Cu_17.5_ HEA under shock loading, contributing to a better understanding of dynamic deformation under extreme environments.

## 1. Introduction

High entropy alloys (HEAs), also known as multi-principal element alloys, have garnered significant attention in the scientific and engineering community due to their exceptional mechanical properties, including ultrahigh strength [1], super-plasticity [2], high temperature stability [3], cryogenic temperature ductility [4], and excellent fracture toughness [5]. In contrast to conventional multicomponent systems, HEAs consist of four or more principal elements with high atomic concentrations (ranging from 5% to 35%). The high conformational entropy of HEAs enables the formation of a simple single-phase solid solution structure [6]. Extensive research was conducted on the deformation mechanism and mechanical properties of HEAs under quasi-static loading [6,7,8,9,10,11]. These studies suggest four principal effects: severe lattice distortion effect [7], high entropy effect [6], sluggish diffusion [8], and cocktail effects [9]. These advantages give HEAs significant potential for utilization in extreme conditions characterized by explosions, ballistic impact, impulsive loadings, and collisions. As a result, obtaining a comprehensive understanding of HEAs’ response to dynamic loading, including deformation mechanisms and failure processes, is imperative to ensure their suitability for extreme conditions, including aerospace, civil transport, and defense domains.

There has been an increase in experimental and computational studies on the dynamic response of HEAs in recent years. Dynamic experiments [12,13,14,15,16,17] were performed using different methods, including split-Hopkinson bars, light-gas guns, and laser shock facilities, to achieve strain rates ranging from 10^3^ to 10^7^/s. For example, in the study conducted by Zhao et al. [12], quasi-static compression and dynamic shearing were applied to the equiatomic CoCrFeMnNi HEA. They observed a dense structure, which consisted of stacking faults, deformation twins, and phase transformations from face-centered cubic (FCC) to HCP, as well as amorphous structures. Yang et al. [16] investigated the shock loading of the Cr_10_Mn_10_Fe_60_Co_10_Ni_10_ HEA, and they observed two phase transformations: from FCC to hexagonal close-packed (HCP) and from FCC to body-centered cubic (BCC) structures. Thürmer et al. [15] studied the shock-induced spallation of the CrMnFeCoNi HEA using a high-power laser. Their results indicated that the deformation mechanism of the material was primarily dominated by nanoscale mechanical twinning at elevated strain rates. Furthermore, their results revealed a high spall strength of approximately 8 GPa at a strain rate of approximately 10^7^/s.

Computational methods such as the molecular dynamics (MD) simulation technique are powerful tools for investigating the effects of shock-induced deformation and damage in HEAs. MD simulation provides valuable insights into the evolution of the material’s microstructure, which is challenging to accomplish with in situ dynamic experiments. For example, the study conducted by Liu et al. [18] investigated the shock-induced dynamic plasticity and failure in FeNiCrCoCu HEAs, suggesting that the plastic deformation mechanisms are influenced by the loading crystalline orientation. In a study by Song et al. [19], the effects of elemental anomaly distribution on the dynamic responses of CoCrFeNiMn alloy are shown, demonstrating that the shock wave propagation, dislocation evolution, defect collapse, and energy dissipation are all dependent on the anomaly arrangement of Mn elements. More recently, Li et al. [20] systematically studied the shock-induced deformation and spallation in CoCrFeMnNi alloy, considering the influence of the crystalline orientation, short-range order, and elements. However, there remains an inadequate microscopic understanding of plastic deformation and phase transformation under shock dynamic loading for HEAs.

Among the numerous HEAs, a single-phase high entropy alloy with non-equal atomic concentrations of FCC Co_25_Ni_25_Fe_25_Al_7.5_Cu_17.5_ was successfully fabricated using mechanical alloying and spark plasma sintering technology [21]. The HEA exhibits exceptional properties, including a superior yield strength of approximately 1.8 GPa and a high hardness of around 454 Hv. These exceptional properties make it suitable for use in shock environments. Computational studies have reported an FCC–BCC phase transformation phenomenon in this HEA under tension loading, indicating that this phase transition can enhance ductility while retaining high strength [22]. The phase transformation is highly dependent on crystalline orientation and strain rate. Furthermore, Wang et al. [23] conducted research on the influence of grain size and Al concentration on the tensile properties of this polycrystalline CoNiFeAl_x_Cu_1-x_ HEA. Chen et al. [24] investigated the compression behavior of both FCC- and BCC-structured polycrystalline CoNiFeAl_x_Cu_1−x_ HEA and reported the Hall–Petch and inverse Hall–Petch relations. Despite extensive research on this particular HEA, there is still limited understanding of its shock response and deformation mechanisms, thereby limiting its application in extreme environments. Specifically, there is a need for further investigation into the impact of phase transformation and crystalline orientation anisotropy on plastic deformation and spall damage under shock loading.

In this study, a series of large-scale molecular dynamics (MD) simulations were conducted to investigate the shock-induced dynamic deformation and spall damage in Co_25_Ni_25_Fe_25_Al_7.5_Cu_17.5_ HEA. The effects of crystalline orientation anisotropy on shock wave propagation, plastic deformation, phase transformation, and spall damage were systematically explored. This paper is organized as follows: Section 2 describes the simulation methodology, Section 3 presents the simulation results and related discussion, and Section 4 summarizes the conclusions.

## 2. Simulation Details

**Figure 1:**
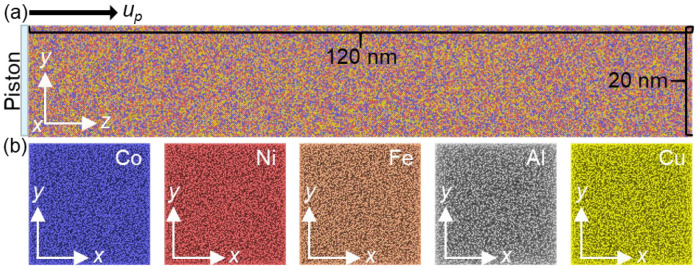
(**a**) Schematic diagram of Co_25_Ni_25_Fe_25_Al_7.5_Cu_17.5_ HEA. The shock wave is generated by a piston with infinite mass with a constant velocity *u_p_*; (**b**) corresponding snapshots of spatial distribution for different elements.

A five-element embedded atom method (EAM) interatomic potential, developed by Zhou et al. [25], was employed to describe the elemental interactions of a nonequal atomic concentration FCC HEA, Co_25_Ni_25_Fe_25_Al_7.5_Cu_17.5_, in this study. Previous studies [23,24] have successfully utilized this potential to investigate the mechanical deformation and FCC–BCC phase transformation of this HEA. Li et al. [22] further demonstrated that the yield strength error between the simulation and experimental values, at a strain rate of 5 × 10^6^/s, was only 4%, highlighting the high accuracy of the current EAM potential. To study the effect of orientation anisotropy, three crystal directions were established: x-[100] y-[010] z-[001], x-[00-1] y-[-110] z-[110], and x-[2-1-1] y-[01-1] z-[111]. Except for crystalline orientation, grain size also plays an important role in the dynamic responses of materials [26,27]. For instance, the wave structure and deformation responses of nanocrystalline Ta exhibit a strong dependence on grain size under shock compression [27]. The flow stress follows the Hall–Petch relation under weak shock intensity but adheres to the inverse Hall–Petch relation under strong shock intensity. Liu et al. [18] discovered that micro-voids tend to nucleate at the grain boundary in nanocrystalline FeNiCrCoCu HEA, resulting in reduced spall strength. Conducting more comprehensive investigations may surpass the scope of this study and can be pursued in future work. In this work, we denote the sample with the *z* direction (shock direction). Figure 1a illustrates the [001] sample, with cell lengths of approximately 20 nm, 20 nm, and 120 nm along the *x*, *y*, and *z* directions, respectively. The system consisted of over 4,000,000 atoms. The *x*-*y* section in Figure 1b shows no apparent element segregation in the HEA, indicating a uniform elemental distribution. The system size is similar to a previous work [18], which is large enough to capture the shock wave propagation and microstructure evolution at the nanoscale.

Prior to shock loading, the system undergoes energy minimization to adjust the atom coordinates using the conjugate gradient algorithm. Afterwards, the configuration is fully relaxed in the isothermal-isobaric ensemble using a Nose–Hoover thermostat and barostat to maintain conditions of 300 K and zero pressure lasting for 50 ps. During relaxation, periodic boundary conditions are maintained in three primary directions. A series of shock simulations were conducted in the micro-canonical ensemble, varying the shock velocity (*u_p_*) from 0.5 km/s to 1.5 km/s. The shock direction is set as the free boundary, while the other directions maintain periodic boundaries to minimize edge effects in the shock waves. The shock wave is generated by an infinite mass plane located at the leftmost position, moving towards the positive *z*-direction. Each simulated shock pulse has a constant duration of 10 ps. After removing the plane, the shock simulations continue for an additional 50 ps. The integration time step used is 1 fs. In order to compare and characterize dynamic responses, the sample is divided into multiple bins along the shock direction to obtain stable stress, particle velocity, temperature, etc. Each bin has a selected thickness of 10 Å. The atomic stress is calculated by the Virial stress formula:

(1) $$\sigma_{\alpha \beta }=-\frac{1}{V}\left(\sum_{i=1}^{N}m_{i}v_{i\alpha }v_{i\beta }+\frac{1}{2}\sum_{i=1}^{N}\sum_{i\neq j}^{N}r_{ij\alpha }f_{ij\beta }\right)$$

where α(β) denotes the x, y or z axis; *V* is the system volume; *N* is the atom’s number; $m_{i}$ and $v_{i}$ represent the velocity and mass of atom *i*; and $r_{ij}$ and $f_{ij}$ are the distance and force vector between atom *i* and atom *j*. The first term indicates the thermal kinetic energy contribution, and the second term arises from the atomic interactions. Note that thermal contribution was calculated by subtracting the translational velocity of the center of mass (COM). Atomic temperature is defined by the formula:

(2) $$T_{i}=(\sum_{j}m_{j}{\left(v_{j}-v_{COM}\right)}^{2}+m_{i}{\left(v_{i}-v_{COM}\right)}^{2})/\left(3(N_{i}+1)k_{B}\right)$$

where *j* goes over the neighbors of atom *i* within r (here *r* is selected as 5 Å); *N_i_* is number of neighbors of atom *i*; *k_B_* is the Boltzmann constant; *m_j_* is the atomic mass of atom *j*; and $v_{COM}$ indicates the COM velocity of the neighbor atoms and is calculated by $v_{COM}=\left(\sum_{j}m_{j}v_{j}+m_{i}v_{i}\right)/\left(\sum_{j}m_{j}+m_{i}\right)$. The average temperature used in a previous study [28] provided better visualization effects compared to the method that directly ignores directional velocity. The maximum shear stress can be calculated using the equation: $\tau ={0.5(\sigma }_{11}-0.5(\sigma_{22}+\sigma_{33}))$, where $\sigma_{11}$, $\sigma_{22}$, and $\sigma_{33}$ represent the principal stress components along *x*, *y*, *z* directions, respectively. This equation has been widely employed in MD simulation works [18,29,30]. All simulations were conducted using the LAMMPS code [31]. The simulation data were visualized and postprocessed using the OVITO code [32] and our self-developed code, MDAPY [33]. The adaptive-common neighbor analysis (a-CNA) method [34] was applied to recognize the atomic microstructure evolution, allowing atoms to be characterized into FCC, BCC, HCP, and other structures. The dislocation extraction algorithm (DXA) [35] was applied to extract the dislocations, which divides the dislocations into Perfect (1/2<110>), Shockley (1/6<112>), Stair-rod (1/6<110>), Hirth (1/3<100>), Frank (1/3<111>) and other type by their Burgers vectors.

## 3. Results

This section discusses the effect of crystalline orientation anisotropy on the dynamic response of HEAs at different shock velocities, using the method of MD simulation. Three main parts are included in this section: (1) plastic deformation during shock compression, (2) spallation damage evolution during the release stage, and (3) estimation of spall strength. The process of shock wave propagation and the plastic deformation mechanism are investigated.

### 3.1. Shock Induced Plasticity

**Figure 2:**
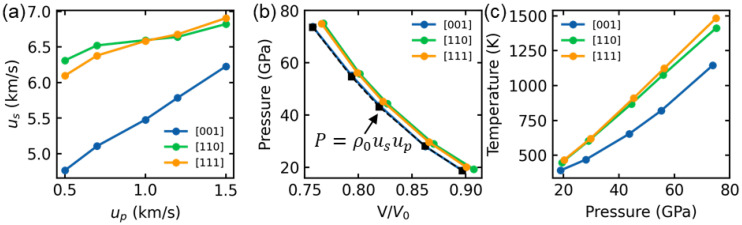
Shock Hugoniot relation for (**a**) shock wave velocity *u_s_* vs. particle velocity *u_p_*, (**b**) pressure vs. relative volume, and (**c**) temperature vs. pressure along [001], [110] and [111] directions, respectively. The black dash line in (**b**) indicates the theoretical Hugoniot pressure curve calculated by the formula: $P=\rho_{0}u_{s}u_{p}$ for [001] sample, where $\rho_{0}$ is the initial density.

Prior to investigating the shock-induced plasticity, we first presented the shock Hugoniot relations for HEA along different crystalline orientations in Figure 2. The shock wave velocity, referred to as the elastic wave speed, is determined by the propagation distance of the elastic wavefront per unit of time. The Hugoniot pressure and temperature are averaged within a 10 nm slice of the post-shocked region. Figure 2a illustrates the relationship between shock wave velocity us and particle velocity up. The results reveal that the shock wave velocities in the [110] and [111] samples are similar to each other and much higher than those in the [001] samples, regardless of the shock intensities. Figure 2b depicts the relationship between Hugoniot pressure and relative volume. The pressure increases from about 18 GPa to 75 GPa. Comparatively, small pressure increases are observed in the [110] and [111] samples compared to the [001] sample at the same shock intensity. This difference is attributed to the variation in slip systems with non-zero Schmitt factors, wherein the [001] sample possesses more such slip systems than the [110] and [111] samples [22]. The presence of fewer of these systems contributes to a lower plastic deformation rate, resulting in a weaker relaxation of stress [18]. Consequently, the Hugoniot pressure is the lowest in the [001] sample and higher in [110] and [111] samples. The theoretical Hugoniot pressure curve for the [001] sample is estimated using the formula: $P=\rho_{0}u_{s}u_{p}$, where $\rho_{0}$ represents the initial density (~7.88 g/cm^3^). The predicted results strongly align with our MD simulation results, thus confirming the accuracy of this study. The shock temperature rises as the shock pressure increases for all loading directions, as depicted in Figure 2c. Nevertheless, at high shock intensity, the discrepancy between the [001] sample and the [110] or [111] sample is greater than that at low shock intensity. This difference is attributed to the development of plastic deformation.

**Figure 3:**
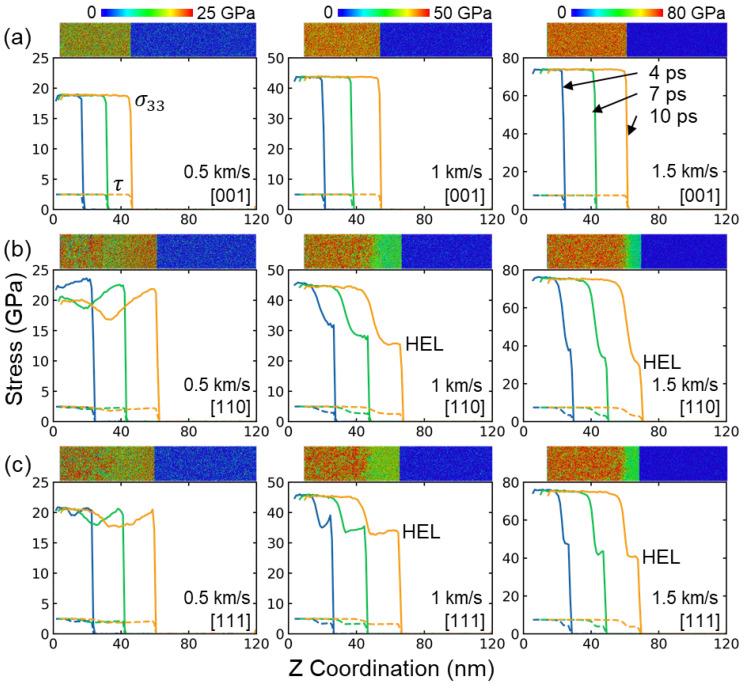
Shock stress $\sigma_{33}$ and shear stress τ profile at several moments under different shock intensities along (**a**) [001], (**b**) [110], and (**c**) [111] directions, respectively. Atoms in the inset microscopic views are rendered by the shock stress. HEL indicates the Hugoniot elastic limit.

Figure 3 presents the profiles of shock stress $\sigma_{33}$ and shear stress τ during the shock compression stage along the [001], [110], and [111] directions. For the [001] sample, the shock stress maintains a platform at approximately 19 GPa (0.5 km/s), 44 GPa (1 km/s), and 74 GPa (1.5 km/s). A similar trend is observed for the variation in shear stress, and the corresponding platform values are approximately 2.5 GPa, 5 GPa, and 7.5 GPa. The atomic stress distribution is uniform in the post-shocked region, which is consistent with the stable stress curve, as shown in the inset views in Figure 3a. In fact, plastic deformation was observed in the [001] sample, which will be discussed in detail in the following part. Consequently, the observed single-wave propagation structure can be attributed to the relatively low elastic wave speed in the [001] sample, and the plastic wave may overlap with the elastic wave, which is consistent with previous research on CoCrFeMnNi HEAs [20]. In terms of the [110] sample, a classical two-zone elastic-plastic wave structure is observed. In the case of 0.5 km/s, both the shock stress and shear stress initially decrease before increasing, as shown in Figure 3b. However, two distinct plateaus are observed for the shock stress and shear stress at higher shock velocities. A similar variation trend is found in the [111] sample (see Figure 3c). For a given stress, the slip systems experiencing the maximum resolved shear stress are likely to be activated. This leads to the orientation dependence of the Hugoniot elastic limit (HEL), which measures the yield strength of a material under the stress state induced by shock loading. In general, the HEL can be determined as the lowest stress value on the plateau of the elastic wave when the elastic precursor wave is separate from the plastic wave [18,36]. The HEL in the [111] sample increases from 17.6 GPa to 40.5 GPa with increasing shock intensity, which is higher than that in the [110] sample (16.8 GPa to 30.8 GPa), as shown in Table 1. Note that experimentally achieving HEL values higher than 10 GPa is challenging. However, in this case, the significant HEL value is attributed to the extremely short propagation length and very high strain rate used in MD simulations [20,37].

**Figure 4:**
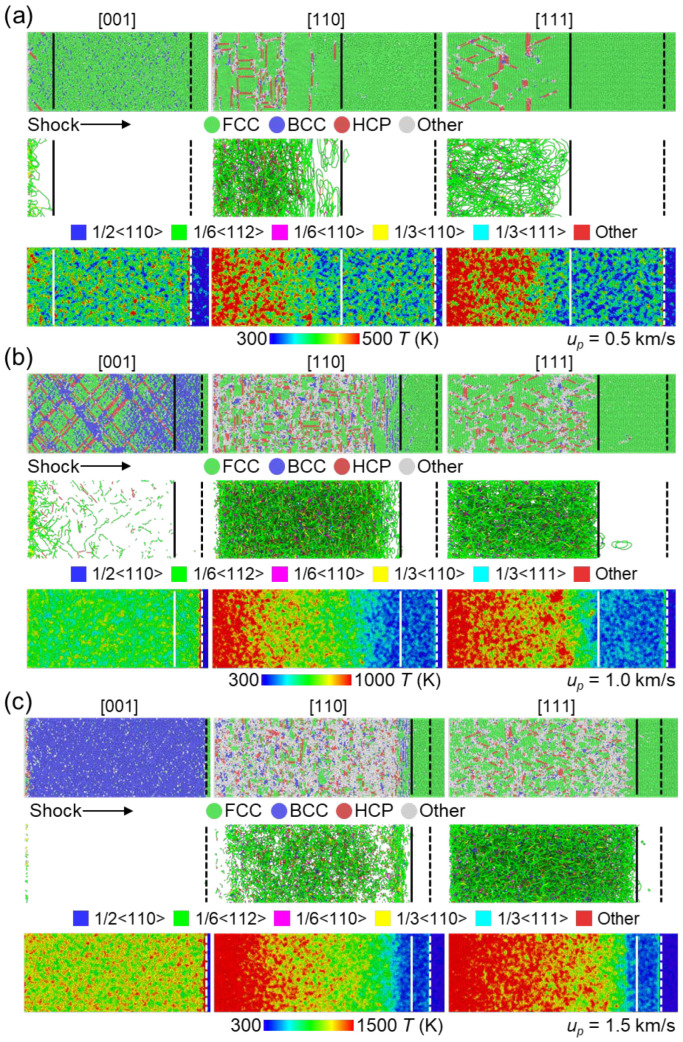
Microstructure observations of the post-shocked regions at 10 ps along the [001], [110], and [111] directions for shock velocities of (**a**) 0.5 km/s, (**b**) 1 km/s, and (**c**) 1.5 km/s, respectively. In each panel, the atoms are visualized based on the structure type determined by the a-CNA method and the atomic temperature. Dislocations are depicted with distinct colors corresponding to their Burgers vector obtained via the DXA method, where atoms are omitted to enhance visualization clarity. The dashed lines represent the elastic wavefront, while the solid lines indicate the position of dislocations.

To investigate the characteristics of shock wave propagation, we have included microstructure snapshots at 10 ps for different shock intensities along the [001], [110], and [111] directions in Figure 4. At a shock velocity of 0.5 km/s, little to no plastic deformation was observed in the [001] sample. Although a few random BCC atoms were present, nucleation of dislocations only occurred near the rare free surface, indicating its instability, as shown in the first column of Figure 4a. On the other hand, noticeable stacking faults (SFs) nucleation (detected as HCP atoms) was observed in the [110] and [111] samples, which extended along the equivalent (111) planes, consistent with a previous work [20]. Additionally, extensive dislocations were found to correspond to the second wave in the two-wave structure. Thus, it can be concluded that the two-wave structure comprises an elastic wave followed by a plastic wave, triggering structural changes in samples with [110] and [111] crystal orientations. In the case of the [001] sample, the temperature distribution was uniform in the post-shocked region, while it exhibited higher temperatures in the plastic deformation region compared to the elastic deformation region in the [110] and [111] samples. When the velocity increased to 1 km/s, stacking faults nucleation along the (111) plane was observed in the [001] sample, with the generation of more BCC phase under shock compression, as shown in Figure 4b. However, no dislocations were detected in relation to the appearance of BCC-structured atoms, suggesting a different deformation mechanism in the formation of BCC-structured atoms. SFs continued to dominate the plastic deformation in the [110] and [111] samples, with the temperature distribution showing a similar trend. Interestingly, a complete transformation from the FCC to BCC structure occurred in the [001] sample at a velocity of 1.5 km/s, which limited the nucleation of SFs and dislocations (see Figure 4c). We will discuss this transformation mechanism in detail in the following part. As for the [110] and [111] samples, apart from the SFs nucleation caused by dislocations, additional disordered structures formed due to the limited number of slip systems unable to rapidly relieve the high shock stress. The low stacking faults energy [22] in these HEAs facilitated the easy nucleation and propagation of 1/6<112> Shockley partial dislocations, which consequently led to the formation of stacking faults under dynamic loading.

**Figure 5:**
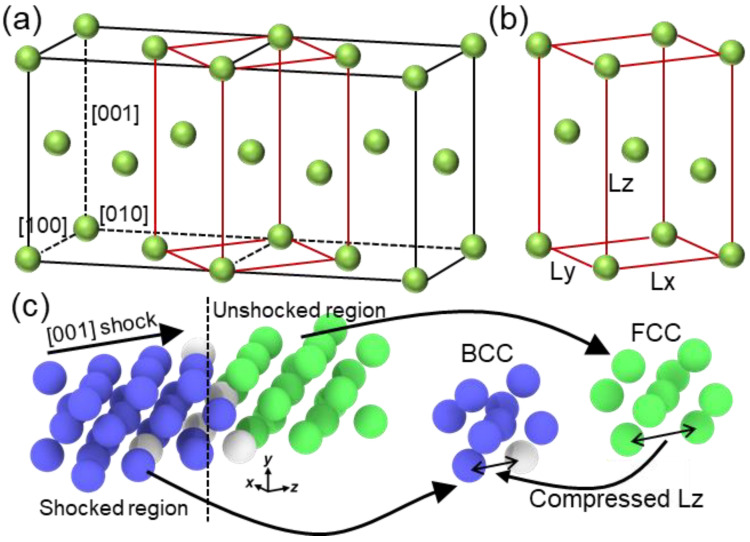
(**a**) Double FCC unit cell; (**b**) body-centered tetragon (BCT) structure in FCC crystal; (**c**) localized atomic snapshot near the shock front at 10 ps for shock velocity of 1.5 km/s along the [001] direction, and the corresponding BCC (blue) and FCC (green) unit cells.

As previously mentioned, the transformation from FCC to BCC does not alter the stress wave structure and generate dislocations. It was reported that the formation of the BCC lattice is linked to the Body-Centered-Tetragon (BCT) structure that arises between two FCC lattices [38], following the Bain path [39]. The FCC to BCC transformation occurs under uniaxial shock compression when the proportions of Lx:Ly:Lz follows the ratio of the $\sqrt{2}:\sqrt{2}$:1, as depicted in Figure 5. This structure change was reported in single-crystal aluminum under shock loading [40], which points out that such strain-induced phase transition differs from the conventional phase transition process. In addition, Xiong et al. [41] observed that the instability of the FCC structure in copper, results in the transformation into the BCC structure, which was explained by applying the modified Born stability criteria and the local minimum energy criterion. Similar phenomena were also found in CoCrFeMnNi HEA [20] and FeNiCrCoCu HEA [18]. Figure 5c illustrates the microstructure near the shock front of the [001] sample with a shock velocity of 1.5 km/s. The shocked region has transformed to the BCC structure while the unshocked region maintains the FCC structure. The corresponding FCC and BCC unit cells exhibit decreased Lz under shock compression. Additionally, the crystalline orientation undergoes changes from [100]||[010]_FCC_ to [110]||[$\bar{1}10$]_BCC_, which is consistent well with the Bain path transformation in aluminum under compression [42]. These findings confirm that the FCC to BCC transformation in the [001] sample for this HEA follows the Bain path under shock compression.

### 3.2. Spallation Damage

**Figure 6:**
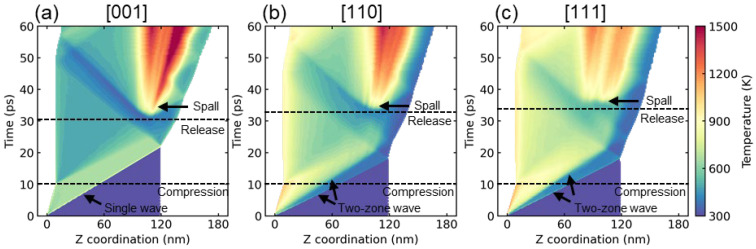
Temperature contours of position-time diagram for (**a**) [001], (**b**) [110], and (**c**) [111] sample at the shock velocity of 1 km/s.

To illustrate the process of shock wave propagation, Figure 6 presents the temperature contours for samples oriented along the [001], [110], and [111] crystallographic directions, with a shock velocity of 1 km/s. The entire process can be divided into three stages: compression, release, and spall fracture. The compression stage is initiated by a piston-induced supported squared wave and lasts for 10 ps. After the piston is removed, an unsupported wave is formed. As the wave reaches the right free surface, a sparse wave is generated and propagates into the sample, resulting in the formation of a tensile region due to the interaction of two opposite sparse waves. The wave in the [001] sample arrives at the surface after 21.8 picoseconds, which is slower compared to the [110] and [111] samples, which arrive at around 18.2 picoseconds. This delay in arrival time can be attributed to the difference in wave speed, as indicated in Figure 2. The release stage continues until void nucleation, which occurs approximately at 30.6, 32.0, and 33.0 ps for the [001], [110], and [111] samples, respectively. Spallation occurs when the maximum tensile stress exceeds the spall strength of the material, and the specific value will be calculated in the subsequent part of this study. In the case of the [110] and [111] samples, a two-zone elastic-plastic wave structure is observed, as shown in Figure 6b,c, consistent with the findings in Figure 3. In all samples, there is a noticeable increase in temperature during spallation, which can be attributed to void nucleation. Furthermore, it is noteworthy that the [001] sample exhibits the highest temperature compared to the [110] and [111] samples.

**Figure 7:**
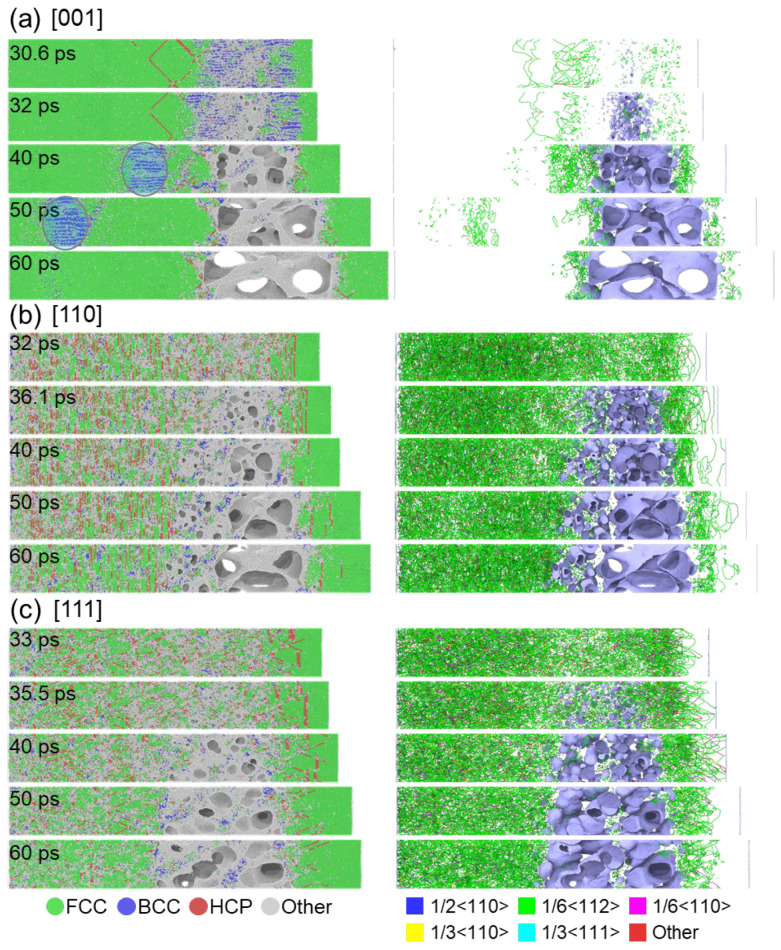
Microstructure snapshots and corresponding dislocation distributions at various time points for (**a**) [001], (**b**) [110], and (**c**) [111] samples at a shock velocity of 1 km/s. Atoms are visualized according to the structure types identified by the a-CNA method. Dislocations are distinguished by their Burgers vectors identified by the DXA method. The pink surface represents voids and is generated using the Construct surface mesh module [43] implemented in OVITO for visualization.

To further investigate the spall damage process, Figure 7 presents the evolution of microstructure and the process of void nucleation for samples oriented in [001], [110], and [111] directions. The shock velocity of 1 km/s is used as an example. Note that the voids are identified using the Construct surface mesh module [43] implemented in OVITO utilizing the alpha-shape method, using a probe sphere with a radius of 0.255 nm, which is the nearest atom distance for HEA. In all samples, spallation occurs as a result of void nucleation, growth, and coalescence, following the classical NAG model [44]. In the [001] sample, a tensile-induced FCC to BCC phase transformation is observed at 30.6 ps, where the void randomly nucleates in the disordered region, as depicted in Figure 7a. This FCC to BCC transformation has previously been reported by Li et al. [22] under uniaxial stress tension loading, indicating that the phase transformation occurs due to the extremely low stacking fault energy and significant lattice distortion that can considerably enhance the strength and ductility of HEA. Moreover, it was found that the phase transformation is influenced by the crystalline orientation and the strain rate. Specifically, such a transformation can occur along the [001] direction but does not occur along the [110] and [111] directions, which aligns well with our findings. Note that the dislocation density in the BCC region is very low, similar to that shown in Figure 4. Additionally, the stacking faults and dislocations in the post-shocked region nearly disappear after the release stage, indicating the reversibility of the FCC to HCP or BCC structural changes. Following the spallation, a new tension region forms to the left of the spall region due to the interaction of sparse waves generated from the fracture surface. Consequently, the BCC phase gradually moves towards the left surface from 40 to 50 ps and disappears at 60 ps due to the release at the surface, as indicated in Figure 7a. However, in the case of the [110] and [111] samples, the stacking faults remain stable even after the release stage (see Figure 7b,c), owing to the limited number of slip systems, which is consistent with the results for the CoCrFeMnNi HEA [20]. The voids nucleate at the intersection regions of the stacking faults and grow with further tensile loading. Additionally, plastic deformation is primarily dominated by Shockley (1/6<112>) dislocations in both the [110] and [111] samples under spall damage.

**Figure 8:**
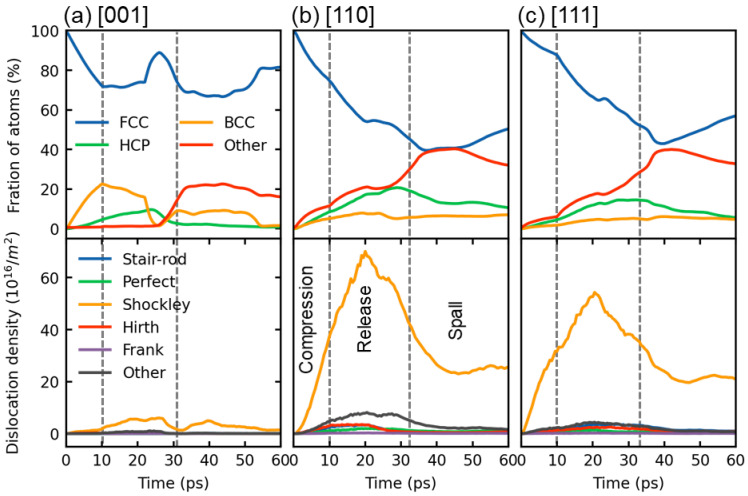
Variation of a fraction of atoms with time for different structures and dislocation density for (**a**) [001], (**b**) [110], and (**c**) [111] samples at a shock velocity of 1 km/s. The dashed lines demarcate the compression, release, and spallation stages of the shock process.

Figure 8 presents a quantitative comparison of the fraction of atom structure types and dislocation density over time for the [001], [110], and [111] directions at a velocity of 1 km/s. In the [001] sample, plastic deformation during the shock compression stage is primarily controlled by stacking faults and the transformation from FCC to BCC, as depicted in Figure 8a. It is noteworthy that the compression strain-induced BCC phase diminishes entirely due to the release effect. However, the interaction of sparse waves creates tensile stress, triggering a transformation from FCC to BCC due to tension-induced strain. Consequently, the BCC fraction restores its growth. The BCC phase remains constant until approximately 50 ps and then vanishes due to the relaxation of tensile stress at the surface. This observation aligns with microstructure views in Figure 7. In contrast, plastic deformation in the [110] and [111] samples is mainly driven by stacking faults, induced by Shockley dislocation motion. Consequently, the [110] and [111] samples exhibit significantly higher Shockley dislocation densities compared to the [001] sample (as presented in Figure 8b,c). Furthermore, the [110] and [111] samples exhibit a substantially greater formation of disordered structures (other atoms) during the release and compression stages due to fewer slip systems, in contrast to the [001] sample, which is consistent with the findings depicted in Figure 7.

### 3.3. Spall Strength

**Figure 9:**
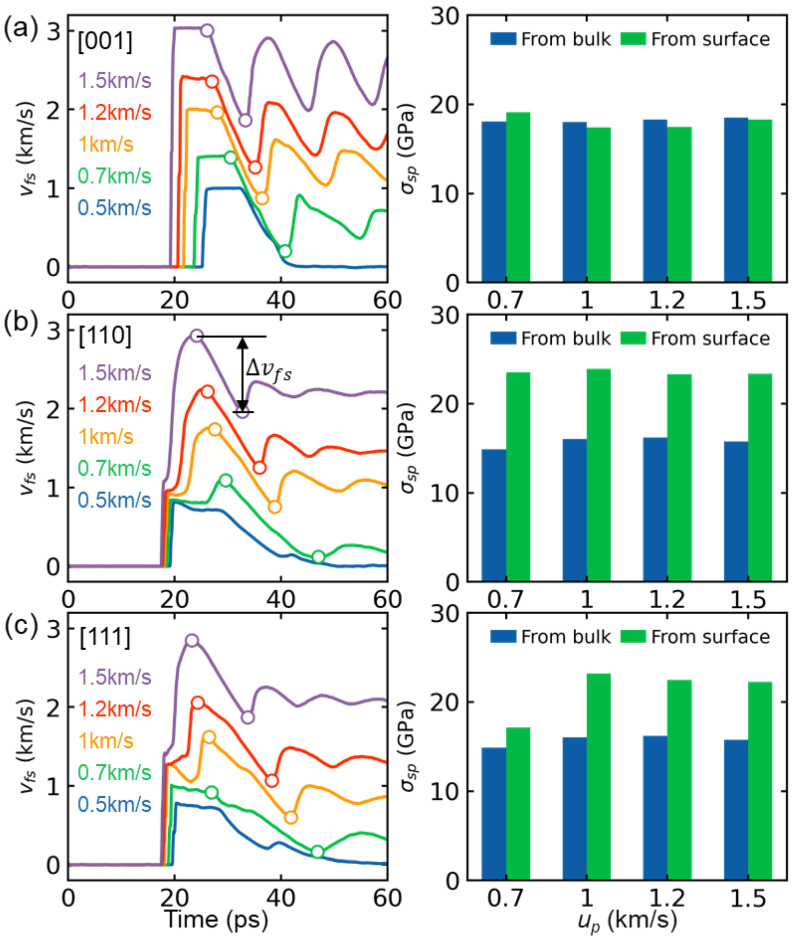
Relationship between free surface velocity $v_{fs}$ and time, along with the corresponding spall strength $\sigma_{sp}$ obtained from bulk and free surface analyses in (**a**) [001], (**b**) [110], and (**c**) [111] samples subjected to varying shock particle velocities ranging from 0.5 to 1.5 km/s. The hollow points are used to determine the $\Delta v_{fs}$.

Gaining a quantitative understanding of the dynamic material strength of HEAs is crucial for evaluating these materials more effectively. In this study, the dynamic tensile strength, represented by the spall strength $\sigma_{sp}$, can be determined either indirectly from the history of the free surface velocity $v_{fs}$ or directly from the bulk material in MD simulations [26]. An indirect method, which relies on the acoustic approximation, is commonly utilized in experiments [45] to measure the spall strength using the formula $\sigma_{sp}=0.5\rho_{0}c_{0}∆v_{fs}$. Here, $\rho_{0}$ represents the material density, $c_{0}$ denotes the sound speed, and $∆v_{fs}$ indicates the pullback velocity in $v_{fs}$ profiles. In this case, $c_{0}$ is estimated from the intercept of the linear fitting for the *u_s_*-*u_p_* curve in Figure 2, resulting in values of 4.059 km/s, 6.132 km/s, and 5.779 km/s for the [001], [110], and [111] samples, respectively. The spall strength from the bulk is determined as the maximum longitudinal tensile stress experienced during the spallation process. Figure 9 illustrates the time-dependent free surface velocity for various shock intensities ranging from 0.5 km/s to 1.5 km/s in the [001], [110], and [111] samples. The pullback velocity acts as an indicator of spallation [46], indicating that no spallation occurs until the shock velocity exceeds 0.7 km/s for all samples. The spall strengths obtained from both the direct and indirect methods are in good agreement for the [001] sample, as depicted in Figure 9a. However, the spall strength derived from the free surface is higher than that obtained from the bulk for the [110] and [111] samples, as shown in Figure 9b,c.

**Figure 10:**
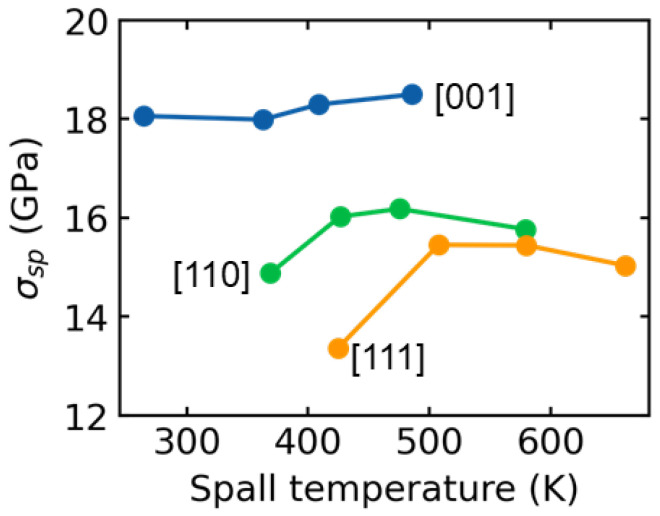
Relation of spall strength $\sigma_{sp}$ obtained from the bulk with the spall temperature for [001], [110], and [111] samples.

We further present the relationship between spall strength obtained from the bulk and the corresponding spall temperature for the [001], [110], and [111] samples, as shown in Figure 10. In this context, the spall temperature is defined as the temperature at which the damaged region reaches its maximum tensile stress. For the [001] sample, the spall strength remains stable as the temperature increases. A similar trend is observed for the [110] and [111] samples when the shock velocity exceeds 1 km/s. This consistent spall strength has also been reported in FeNiCrCoCu HEA [18], with shock velocities ranging from 0.7 km/s to 1.2 km/s. It is found that the [001] sample exhibits the highest spall strength compared to the [110] and [111] samples for all shock intensities, which can be attributed to the FCC to BCC transformation during the tension stage [22], as shown in Figure 7. Our previous work [47] also demonstrates that the ultimate strength for this HEA along [001] direction is higher than that along [110] and [111] directions under uniaxial strain tension loading at a strain rate of 10^9^/s, which is highly consistent with the present results. These results demonstrate a significant anisotropy of crystalline orientation in the dynamic responses of HEAs.

## 4. Conclusions

In this study, the shock response and dynamic failure of single-crystal Co_25_Ni_25_Fe_25_Al_7.5_Cu_17.5_ HEA are investigated using large-scale MD simulations, examining the behavior of the HEA when exposed to shock velocities ranging from 0.5 to 1.5 km/s. The shock pressure increases from around 18 GPa to 75 GPa. The results reveal that the plastic deformation mechanisms of the HEA are highly dependent on crystallographic directions. Specifically, the shock wave velocity in the [110] and [111] direction is significantly higher than that in the [001] direction. This difference in shock wave velocity leads to the observation of a typical two-zone elastic-plastic shock wave structure in the [110] and [111] samples, whereas only a single-wave structure is found in the [001] sample. Under shock compression in the [001] direction, the plasticity deformation is primarily dominated by a transformation from FCC to BCC following the Bain path, as well as dislocation slip. On the other hand, in the [110] and [111] directions, the plasticity deformation is only attributed to dislocation slip. During the release stage, the stacking faults and BCC phases disappear in the [001] sample, while the [110] and [111] samples retain a significantly higher density of stacking faults and dislocations. The main dislocation type is the Shockley dislocation. The spall strength, which is the resistance to spall fracture, is determined by comparing the values obtained from bulk and free surface velocity profiles. The results show reasonable consistency. Furthermore, the spall strength remains stable with increasing shock intensity, and the [001] sample exhibits higher spall strength (~18 GPa) compared to the [110] (~16 GPa) and [111] (~15 GPa) samples. This is attributed to the tension strain-induced transformation from the FCC to the BCC phase, which enhances the strength of the material. Overall, these findings provide important insights into the shock response of Co_25_Ni_25_Fe_25_Al_7.5_Cu_17.5_ HEAs and offer valuable references for potential applications in extreme conditions.

## Figures and Tables

**Table 1:** HEL under different shock velocities along [110] and [111] directions.

	0.5 km/s	0.7 km/s	1 km/s	1.2 km/s	1.5 km/s
[110]	16.8 GPa	20.6 GPa	25.3 GPa	27.6 GPa	30.8 GPa
[111]	17.6 GPa	23.8 GPa	32.6 GPa	32.8 GPa	40.5 GPa

## Data Availability

The data presented in this study are available on request from the corresponding author.
